# Envisioning the Future of Personalized Medicine: Role and Realities of Digital Twins

**DOI:** 10.2196/50204

**Published:** 2024-05-13

**Authors:** Alexandre Vallée

**Affiliations:** 1 Department of Epidemiology and Public Health Foch Hospital Suresnes France

**Keywords:** digital health, digital twin, personalized medicine, prevention, prediction, health care system

## Abstract

Digital twins have emerged as a groundbreaking concept in personalized medicine, offering immense potential to transform health care delivery and improve patient outcomes. It is important to highlight the impact of digital twins on personalized medicine across the understanding of patient health, risk assessment, clinical trials and drug development, and patient monitoring. By mirroring individual health profiles, digital twins offer unparalleled insights into patient-specific conditions, enabling more accurate risk assessments and tailored interventions. However, their application extends beyond clinical benefits, prompting significant ethical debates over data privacy, consent, and potential biases in health care. The rapid evolution of this technology necessitates a careful balancing act between innovation and ethical responsibility. As the field of personalized medicine continues to evolve, digital twins hold tremendous promise in transforming health care delivery and revolutionizing patient care. While challenges exist, the continued development and integration of digital twins hold the potential to revolutionize personalized medicine, ushering in an era of tailored treatments and improved patient well-being. Digital twins can assist in recognizing trends and indicators that might signal the presence of diseases or forecast the likelihood of developing specific medical conditions, along with the progression of such diseases. Nevertheless, the use of human digital twins gives rise to ethical dilemmas related to informed consent, data ownership, and the potential for discrimination based on health profiles. There is a critical need for robust guidelines and regulations to navigate these challenges, ensuring that the pursuit of advanced health care solutions does not compromise patient rights and well-being. This viewpoint aims to ignite a comprehensive dialogue on the responsible integration of digital twins in medicine, advocating for a future where technology serves as a cornerstone for personalized, ethical, and effective patient care.

## Introduction

Digital twins have emerged as a groundbreaking concept in the field of personalized medicine, offering immense potential to transform health care delivery and improve patient outcomes [1-3]. By creating digital replicas of individuals and leveraging advanced technologies, digital twins enable health care professionals to gain a comprehensive understanding of patients’ health, personalize treatment plans, and make data-driven decisions [4,5]. In this era of rapid technological advancements, digital twins have proven to be instrumental in enhancing the understanding of patient health, predictive modeling, risk assessment, digital clinical trials, remote patient monitoring, and telemedicine [6,7].

One of the key advantages of digital twins is their ability to integrate and analyze diverse data sets, including electronic health records (EHRs), wearable devices, genetic information, and patient-reported data [8]. This comprehensive data integration empowers health care providers to have a holistic view of a patient’s health status, identifying patterns, correlations, and potential health risks that might go unnoticed with isolated data sources. Through real-time monitoring and feedback, digital twins enable continuous assessment of a patient’s health, allowing for timely interventions and adjustments to treatment plans when necessary [1,6].

Digital twins also have a major role in predictive modeling and risk assessment [9]. By leveraging artificial intelligence (AI) algorithms and machine learning techniques, digital twins can simulate disease progression [10], predict treatment responses [11,12], and conduct personalized risk assessments [13]. This personalized approach considers factors such as genetics, lifestyle choices, and environmental influences, enabling health care providers to offer targeted preventive measures, early detection strategies, and personalized interventions to mitigate identified risks [14,15].

Moreover, digital twins have the potential to revolutionize digital clinical trials and drug development [2]. By creating digital patient populations and simulating treatment effects, digital twins streamline the drug discovery process, optimize trial design, and reduce costs and timelines [16,17]. They enable researchers to assess the efficacy and safety of drug candidates before conducting traditional trials, accelerating the availability of new treatments to patients [16].

Moreover, digital twins contribute significantly to remote patient monitoring and telemedicine [18]. By leveraging wearable sensors, Internet of Things (IoT) devices, and remote data transmission, digital twins facilitate continuous monitoring of patient health parameters, personalized interventions, and remote consultations [13]. This approach improves patient access to health care services, reduces hospitalizations, and empowers individuals to actively participate in their own health care management [19].

As the field of personalized medicine continues to evolve, digital twins hold tremendous promise in transforming health care delivery and revolutionizing patient care [20]. By harnessing the power of data integration, predictive modeling, digital simulations, and remote monitoring, digital twins enhance the understanding of patient health, optimize treatment strategies, and pave the way for a more personalized and efficient health care system [1]. Digital twins are enhancing health care by providing health care professionals with a deeper understanding of patients’ health, enabling personalized care, and optimizing treatment strategies. They also hold immense potential in advancing drug development and making health care more accessible through remote monitoring and telemedicine. Personalized medicine is at a crossroads, and digital twins represent a path toward a more precise and patient-centric health care paradigm. Drawing from years of research and clinical observations, it is important to argue that the integration of digital twins is not just an advancement but a necessity for modern health care. Thus, this viewpoint critically examines the role of digital twins on personalized medicine across (1) the understanding of patient health, (2) risk assessment, (3) clinical trials and drug development, and (4) patient monitoring.

## Digital Twin

In recent years, the concept of digital twins has been receiving increasing attention from both researchers and engineers. As research in the field of digital twins progresses, carried out by both industry and academia, the boundaries between digital twins and other related concepts have started to blur. Initially, the scope of the digital twin included physical and digital products along with their interconnections [21]. This concept has evolved due to the rapid advancements in communication technology, sensor technology, big data analysis, IoTs, and simulation technology [22]. This growth has led to significant research into digital twins, even if digital twins remain theoretical applications.

Subsequently, the digital twin was redefined as a digital replication of living or nonliving physical entities, opening applications in areas such as health and well-being [23]. As a dynamic concept, the digital twin represents a digital replica of human organs, tissues, cells, or microenvironments that continuously adapts to real-time data variations and predicts corresponding future scenarios [24]. However, the digital twin goes beyond being just a digital model linked to its real-life counterpart through emerging technologies. It emerges as a sentient, intelligent, and evolving model capable of optimizing processes and continually forecasting future states, such as defects, damages, and failures, through a closed-loop interaction between the digital twin and its surrounding environment.

Broadly, the technologies crucial for the digital twin can be categorized into 2 groups as follows: one involves a data-driven statistical model, while the other integrates multiscale knowledge and data into a mechanical model [25]. The numerical model calculates structural performance, while the analytical model facilitates structural analysis. An AI model, trained with samples and numerical data, derives real-time structural insights from sensor data.

The impact of the digital twin is profoundly reshaping industries and has been adopted by major corporations to enhance efficiency and identify issues. This transformative technology is also making its way into the health care sector. In this context, the digital twin can treat patients as digitalized stand-alone assets applicable to various health care scenarios [26]. This potential holds significant promise for improving treatment and diagnostics within hospitals and for individual patients.

A digital twin represents a digital replica of a tangible entity or process, such as a patient, their anatomical structure, or the setting of a hospital. Currently, digital twins are designed to dynamically mirror various data sources, including disease registries, “-omics” data (such as genomics, biomics, proteomics, or metabolomics data), as well as physical indicators, demographic information, and lifestyle data pertaining to an individual’s progression over time [27]. The evolution of foundational technologies like the IoTs and AI, coupled with the availability of an expanding array of accurate and accessible data types (ranging from biometric and behavioral data to emotional, cognitive, and psychological insights), has sparked increased interest and exploration in the research and potential applications of digital twins within the health care domain [27].

## Enhanced Understanding of Patient Health

Digital twins have a major role in providing health care professionals with an in-depth and comprehensive understanding of an individual’s health status [28]. By integrating and analyzing data from various sources, digital twins offer a holistic view of a patient’s physiological parameters, lifestyle patterns, and genetic predispositions [21,29]. This enhanced understanding enables health care providers to personalize treatment plans and interventions according to an individual’s unique needs, ultimately improving patient outcomes [30].

Digital twins integrate data from multiple sources, including EHRs [5], wearable devices [24], genetic information [31], and patient-reported data [2]. This comprehensive data integration allows health care professionals to gain a more complete picture of a patient’s health. By aggregating and analyzing diverse data sets, digital twins can identify patterns, correlations, and potential health risks that might not be evident through isolated data sources [32].

Digital twins enable continuous real-time monitoring of patient data, providing health care providers with up-to-date information on vital signs, biomarkers, medication adherence, and other relevant parameters [33]. This real-time feedback allows for immediate assessment of a patient’s health status and the ability to make timely interventions or adjustments to treatment plans when necessary [34]. Real-time data can be provided by IoT solutions, and large data flows may be managed and secured by robust digital infrastructures [6]. For example, a hospital’s digital transformation team has put forth a plan to create an advanced decision support model that uses real-time data from various health care systems and devices. This model facilitated the evaluation of the effectiveness of current health care delivery systems and the assessment of the potential impact of service modifications, all while seamlessly integrating with the hospital’s day-to-day operations. It offered the ability to predict the outcomes of proposed model changes before implementing them in practice [35].

By leveraging AI algorithms and machine learning techniques, digital twins can analyze patient data to assess an individual’s risk factors for specific diseases or health conditions with high levels of performance [31,36] (Table 1). This personalized risk assessment considers a patient’s genetic profile, lifestyle choices, environmental factors, and other relevant data [37,38]. With this information, health care providers can offer targeted preventive measures, early detection strategies, and personalized interventions to mitigate the identified risks.

**Table 1:** Models and accuracies of digital twins in research.

Year	Applications	Modeling	Database	Accuracy	Reference
2022	Cancer analyses progression and specific organ identification	Natural language processing labeling with CNN^a^, recurrent neural network models, term frequency-inverse document frequency ensemble model	Computed tomography scans (2009-2021)	In validation population: 93.8% for lung cancer, 92.5% for liver cancer, and 96.1% for adrenal cancer with term frequency-inverse document frequency ensemble model; 96.8% for lung, 99% for liver, and 99.7% for adrenal with augmented CNN.	[39]
2022	Personal health care improvement with emotion recognition	Automatic detection of emotion from EEG^b^ signals with gradient boosting, k-nearest neighbor, and RF^c^ models	EEG images	99.9% with gradient boosting, 98.6% with decision tree classifier, 99.7% with k-nearest neighbor, and 99.6% with RF	[40]
2022	Prostate cancer progression with biochemical recurrence and seminal vesicle	Machine learning methods with support vector machine, RF, NN, recurrent neural network, and long short-term memory models	Clinical data warehouse with patients with cancer	82.7% of accuracy for biochemical recurrence and 83.9% for seminal vesicle	[41]
2021	Abdominal aortic aneurysm severity detection	Inverse analysis with CNN and long short-term memory models	Digital patient’s data set	99.91% for detection and 97.79% for severity	[42]
2021	Prevention of stroke and treatment of poststroke	Support vector machine	EEG data set	76% accuracy and 0.84 for performance	[43]
2019	Fault diagnosis pattern	Deep neural network model with deep-neural network and deep transfer learning (DFDD^d^)	Digital patient’s data set	98% for accuracy with DFDD and 91.5% with deep-neural network	[44]
2019	Ischemic heart disease detection	Deep neural model	Pulmonary tuberculosis diagnostic electrocardiogram database	85.8% for the implemented model	[45]

^a^CNN: convolutional neural network.

^b^EEG: Electroencephalography.

^c^RF: random forest.

^d^DFDD: fault diagnosis method using deep transfer learning.

Digital twins can simulate and predict the progression of diseases based on real-time patient data [2]. By using historical patient data and integrating it with the current health parameters, digital twins can generate predictive models that help anticipate disease progression, identify potential complications, and estimate treatment outcomes [46]. This predictive modeling enables health care professionals to make informed decisions and develop personalized treatment plans that maximize efficacy and minimize risks.

Moreover, digital twins facilitate longitudinal tracking of patient health data, allowing health care providers to analyze trends and changes over time. This longitudinal view provides insights into disease progression, response to treatments, and the impact of lifestyle modifications on overall health outcomes [47,48]. Thus, by identifying the patterns and trends, digital twins can help health care professionals identify personalized interventions and adjustments to optimize patient care [1].

## Predictive Modeling and Risk Assessment

Digital twins can leverage advanced algorithms and machine learning techniques to perform predictive modeling and risk assessment in the field of personalized medicine [49]. By analyzing real-time patient data and historical records, digital twins enable health care professionals to anticipate disease progression, identify potential risks, and optimize treatment strategies [2].

Digital twins can simulate and predict the progression of diseases based on patient-specific data [19]. By integrating various data sources such as genetic information, biomarkers, lifestyle factors, and treatment history, digital twins create digital replicas of patients and model the progression of diseases over time [5]. This modeling allows health care professionals to forecast potential outcomes, anticipate complications, and adjust treatment plans accordingly.

Digital twins enable health care providers to predict how individual patients will respond to different treatment options [21,50]. By analyzing data from similar patient cases and incorporating real-time patient data, digital twins can generate predictive models, taking into consideration uncertainty and confidence intervals, that estimate treatment outcomes [46,51]. This information helps health care professionals make informed decisions about the most effective treatments for individual patients, increasing treatment success rates and reducing trial and error [52,53].

Digital twins assess individual patients’ risks for specific diseases or health conditions by analyzing their genetic profiles, lifestyle choices, environmental factors, and other relevant data [1,28]. By integrating this information and leveraging machine learning algorithms, digital twins can identify personalized risk factors and quantify the likelihood of developing certain conditions [54]. This personalized risk assessment enables health care providers to implement preventive measures, early detection strategies, and targeted interventions to mitigate risks [55].

Furthermore, digital twins can serve as early warning systems by continuously monitoring patient data and identifying potential health risks or deviations from normal patterns [56,57]. By establishing baseline parameters for each patient, digital twins can detect anomalies or warning signs that may indicate the onset of a disease or adverse health event [58]. This early detection allows for timely interventions, proactive health care management, and prevention of complications [1]. By considering individual patient characteristics, such as genetics, lifestyle, and response to previous treatments, digital twins can recommend tailored treatment plans that maximize efficacy and minimize side effects [5,21]. This personalized approach helps health care professionals select the most suitable treatments, dosages, and interventions for each patient, leading to improved outcomes.

The use of digital twins for predictive modeling and risk assessment presents several ethical considerations that should be carefully examined and addressed [59]. The collection and storage of data should be done with a strong focus on privacy and data security and ensuring that personal or sensitive information is adequately protected is paramount [57]. Moreover, individuals whose data are used in digital twin models should provide informed consent. They should be aware of how their data are being used and could opt out if they choose [21]. Furthermore, there should be transparency in how digital twins are created and used. Clear explanations of the modeling process and the factors influencing predictions should be provided to stakeholders, including regulators and the public [59].

## Digital Clinical Trials and Drug Development

Digital twins are poised to revolutionize the landscape of clinical trials and drug development, offering significant advantages over traditional approaches [60]. By simulating digital patient populations and leveraging advanced technologies, digital twins streamline the drug discovery process, accelerate clinical trials, and enhance the efficacy and safety of new treatments [60].

The creation of digital patient populations that closely resemble real-world patient demographics is now possible with the future development of digital twins [61-63]. These digital populations can be customized to reflect diverse characteristics, such as age, sex, genetics, and comorbidities [30]. By generating digital patients with varying profiles, digital twins provide a comprehensive representation of the population under study, improving the generalizability of trial results [64]. Indeed, digital twins could simulate the effects of potential treatments on digital patients, considering individual patient characteristics and treatment responses [51]. This allows researchers to assess the efficacy and safety of drug candidates before conducting costly and time-consuming clinical trials. Digital twins assist in optimizing the design of clinical trials by providing insights into patient recruitment, trial end points, sample size determination, and treatment protocols [12,27]. By analyzing digital patient populations, digital twins can predict the likely response rates, treatment effects, and potential adverse events, helping researchers tailor trial parameters for maximum efficiency and statistical power [65,66]. Digital clinical trials conducted through digital twins offer substantial cost and time savings [51]. By replacing or supplementing traditional trials, which often involve extensive site visits and patient recruitment, digital twins streamline the data collection process. Additionally, digital trials eliminate the need for physical infrastructure, reduce administrative burdens, and enable remote patient monitoring [67]. These efficiencies translate into reduced trial costs and shorter timelines, expediting the availability of new treatments to patients in need [60].

The contribution of such tools can improve patient safety in drug development [16]. By using digital patient data, digital twins can assess the potential risks and side effects of new treatments, enabling researchers to optimize dosages and identify vulnerable patient subgroups [16]. This approach minimizes the exposure of actual patients to experimental treatments, ensuring ethical considerations and protecting patient well-being [57,68].

The facilitation of real-time monitoring of digital patients by digital twins can allow researchers to collect and analyze data continuously [69-71]. This capability enables adaptive trial designs, where trial parameters can be modified based on ongoing analysis of digital patient responses [72,73]. Adaptive trials enhance the efficiency of clinical research by reducing the number of patients required, optimizing treatment regimens, and maximizing the chances of success [74].

Clinical drug development is an intricate and time-intensive journey, spanning roughly 6-15 years [75,76]. The financial commitment for shepherding a novel drug from its conceptual infancy through the labyrinth of research, development, and eventual market approval teeters at an astounding sum of approximately US $2.6 billion [77]. Shockingly, approximately 85% of potential therapies stumble in the early stages of clinical development, and of the fortunate few that reach phase 3, only half earn the coveted stamp of approval [78]. Another daunting statistic reveals that nearly 80% of trials falter in their attempts to meet their initial enrollment goals and prescribed timelines, which translates into a staggering daily revenue loss of up to US $8 million for pharmaceutical behemoths [75]. Furthermore, an annual expenditure nearing US $6 billion is dedicated to the pursuit of patient recruitment. Remarkably, merely 2% of the eligible population in the United States partakes in these clinical odysseys and those brave souls who do so endure an average of 11 visits to the trial site within the span of 6 months [79].

From an economic standpoint, there looms the tantalizing prospect of savings entwined with digital clinical trials. This progressive approach is geared toward streamlining the study duration, curtailing the recruitment period, and accelerating the data collection process. Furthermore, it obliterates the conventional need for numerous physical study sites, offering an alluring window into substantial capital savings, with estimated costs of managing a solitary digital clinical trials study site fluctuating between US $1500 and US $2500 per month [75]. Additionally, the demand to reimburse patients for their travel-related expenses dwindles, providing an enticing financial incentive. However, it is imperative to acknowledge that this paradigm shift is contingent upon robust technological support, which is the cornerstone upon which these cost-saving benefits are built.

The creation of digital populations brings forth a fresh perspective, coupled with inventive techniques and methodologies. Over recent years, the concept of generating digital populations through data augmentation of existing data sets has been steadily gaining traction. One notable example is the Synthea project [80], which simulates EHR data based on the fundamental demographics of the Massachusetts population and specific disease models. In a similar vein, endeavors have been made to craft digital patients with precise measurements, such as glucose levels, either through mathematical models [62] or by incorporating highly specific attributes [81]. A digital patient data set is constructed by leveraging real-world data, including demographics, laboratory findings, and anatomical features. Subsequently, using patient-specific disease progression models, diverse vascular models can be generated and explored [82].

In situations where an extensive patient data set is available, clinical trial simulations necessitate the careful selection of a representative subset from the original pool. However, when dealing with limited data sets, the imperative arises to augment the existing data. This process, which introduces digital patients into the original data set, serves as an invaluable technique. Its reliability shines through, particularly in data sets characterized by non-Gaussian distributions of covariates [83].

Conventional clinical trial designs often overlook the intricate patient diversity and intricacies. The inherent heterogeneity of patients enrolled in clinical trials manifests as a correspondingly wide spectrum of responses to stent implantation. Enter the era of digital populations applied to the realm of novel stent design, poised to enhance patient safety, slash the costs of clinical trials, and ultimately usher in a new era of clinical practice [84]. Thus, highly efficient stents with minimized side effects, promise a brighter future for patients.

## Remote Patient Monitoring and Telemedicine

Digital twins can enable remote patient monitoring and telemedicine, revolutionizing the way health care is delivered and accessed [21,85]. By leveraging wearable sensors, IoT devices, and advanced technologies, digital twins enable health care providers to remotely monitor patients, offer timely interventions, and enhance the quality of care [86-88]. Thus, digital twins facilitate continuous monitoring of patient health parameters, regardless of the patient’s location. By integrating data from wearable devices, sensors, and other IoT-enabled devices, digital twins collect real-time information on vital signs, medication adherence, and other relevant health data. This continuous monitoring enables health care providers to stay informed about a patient’s condition, detect anomalies, and intervene promptly when necessary [6,89].

Digital twins enable the seamless transmission of patient data from remote locations to health care providers [23]. Through secure data channels, patient information is sent to the digital twin, which can analyze and interpret the data in real time [90]. This remote data analysis provides health care professionals with valuable insights into a patient’s health status, allowing for remote assessment and decision-making [64,91]. By analyzing the data collected through remote monitoring, digital twins can also identify trends, patterns, and potential health risks specific to an individual patient [28,68]. This information enables health care professionals to tailor interventions, adjust treatment plans, and provide personalized guidance to patients, enhancing the effectiveness and efficiency of care delivery.

Remote patient monitoring and telemedicine facilitated by digital twins can help reduce hospitalizations and readmissions [92,93]. By closely monitoring patients’ health at home or in nonacute care settings, health care providers can detect early signs of deterioration or complications. Timely interventions can then be initiated, preventing the need for hospitalization, or reducing the length of hospital stays. This approach also supports the transition from hospital to home, ensuring continuity of care and reducing the likelihood of readmissions [5,6].

The promotion of patient engagement and self-management could be enhanced by digital twins by empowering individuals to take an active role in their own health care. By providing access to their own health data, patients can monitor their progress, track their vital signs, and gain insights into their health conditions [2,27,85]. Digital twins can also deliver personalized recommendations, reminders, and educational materials to patients, supporting self-care and adherence to treatment plans [21,24]. Through videoconferencing, secure messaging platforms, and real-time data sharing, digital twins can facilitate digital visits, allowing patients to consult with health care professionals from the comfort of their homes [51]. This remote access to health care services improves convenience and accessibility, and reduces the need for physical appointments, particularly for patients in rural or underserved areas.

Digital twins support long-term disease management by providing continuous monitoring and personalized interventions [56,64]. For patients with chronic conditions, digital twins enable health care providers to remotely track disease progression, assess treatment effectiveness, and adjust therapies as needed. This proactive approach helps patients maintain optimal health, prevent complications, and reduce the burden of frequent hospital visits.

Conversely, a pivotal challenge in the implementation of digital twins lies in the absence of seamless connectivity among various systems and medical devices within the Digital Health Twin framework. The solution to this quandary lies in the widespread adoption of standardized data representation and exchange protocols. These protocols serve as the linchpin for facilitating smooth communication and the harmonious integration of EHRs, medical devices, and assorted health care systems [94]. This newfound interoperability, in turn, fosters the exchange of patient-specific information, elevating the precision and comprehensiveness of the DHT model [95].

The successful implementation of digital twins necessitates a tapestry of expertise, a multidisciplinary symphony where physicians, radiologists, image processing virtuosos, molecular biologists, geneticists, bioinformaticians, computer scientists, data wizards, and engineers converge. Their collective efforts are pivotal in confronting the intricate challenges inherent in crafting a digital counterpart that is both accurate and dependable [96].

## Challenges for Digital Twin Implementation

The digital twin market faces formidable challenges that could impede its growth (Table 2). These obstacles encompass the high deployment costs, surging demands for power and storage, integration issues with existing systems and proprietary software, as well as the intricate nature of its architecture. The implementation of digital twin solutions is a costly endeavor, necessitating substantial investments in technology platforms (comprising sensors and software), infrastructure development, maintenance, data quality control, and security solutions. Digital twin constantly collects, analyses, and accumulates data from physical space to provide sufficient information for decision-making. Thus, the challenge of data integrity and security remains major. This includes measures like data encryption, secure data storage, and regular backups for privacy data used by digital twin models. By real-time data perception of dynamic environment and high accuracy model, digital twin should include regular control processes for performance prediction. Moreover, the upkeep of the digital twin infrastructure incurs significant operational expenses. The high fixed costs and the complexity of digital twin architectures are anticipated to decelerate the adoption of digital twin technologies. Digital twins pose a formidable challenge in their demand for rich, extensive data sets and innovative EHR designs that facilitate data mining and the automated acquisition of pristine data. Currently, one of the major impediments to human digital twins is the glaring heterogeneity and operational intricacies found in EHRs and health care information systems [12]. Furthermore, these data often reside in an unstructured format, necessitating either manual intervention or the deployment of advanced automation through natural language processing technologies to extract the required information.

**Table 2:** Challenges for digital twin implementation for personalized medicine.

Challenge	Description
Challenge 1	Methodological hazards were a noted challenge to using artificial intelligence for inductive reasoning; for example, the generalizability of findings necessitates external validation with new patient cohorts, or cohorts from different centers or different geographical locations, and across time.
Challenge 2	The Internet of Things as applied to health care presents challenges for devices with processor, memory, and energy limitations.
Challenge 3	Transformational technologies present demands on digital twin software for confidentiality, reliability, safety, and secure coding, with minimal requirements for patching.
Challenge 4	The credibility of digital patient models to predict disease risk and progression in a real patient, and the trust required of the computational processes that deliver these, presents a potential barrier to their uptake into a routine workflow.
Challenge 5	Creating a digital twin of a patient for precision medicine raises considerable ethical questions around its legacy, privacy, and identity; and its termination when the real twin dies.
Challenge 6	Potential regulatory and legal issues for a health digital twin are yet uncertain but are likely to be especially demanding for approval of devices associated with medical cyber-physical systems that contain large amounts of embedded software for sensing and monitoring people’s activities.

The quality of the data plays a paramount role. Although sensors are adept at efficiently collecting and transmitting data to human digital twins, the processes involved in gathering hospital data can be both expensive and time-consuming [96]. Presently, many individual data are procured through blood tests, imaging systems, and health scans. Consequently, these hospital data collection procedures place a considerable burden on digital twin processes. For instance, achieving top-tier image quality in computerized tomography scans of cardiac patients is no straightforward task, and the results often hinge on the expertise of radiology personnel, especially those with limited experience. Experts in the field argue that the future milestones in digital twins will not revolve around the advancements in AI research but rather focus on rectifying the issues associated with small-scale, unorganized health care data [12].

While digital twin applications have been portrayed as fully autonomous processes, there is an essential need for interdisciplinary knowledge spanning fields such as biomedicine, mathematics, bioengineering, and computer science, as well as insights derived from people’s experiences, given the intricate nature of human beings [96]. Moreover, digital twin software developers should prioritize the creation of user-friendly interfaces for digital twins to facilitate communication between digital twin software, patients, and physicians. These interfaces should enable discussions on optimal treatment based on informed consent. Nevertheless, experts in the field have identified a dearth of user-friendly software for digital twin applications in the realm of health care [97].

Physicians continue to harbor reservations about placing trust in decisions derived from algorithms and big data, primarily because these predictions often lack a plausible or transparent explanation. Recent research on the integration of AI systems in the hospital environment reveals that many physicians remain skeptical of AI due to the significant risk associated with potential misdiagnoses and inappropriate treatment decisions. The apprehension of clinicians being replaced may surface with the broader use of digital twins in clinical tasks [12]. In certain scenarios, digital twins may surpass clinicians in performance, as it is highly improbable that a clinician could process all patient data and provide a solution during a brief consultation. Nonetheless, given the prevailing mistrust of digital twin decision points and the current state of digital twin applications in health care, digital twins will strive to adapt to the needs and workflows of clinicians in the future. This adaptation aims to enhance their capacity to efficiently consider the entire spectrum of available information when making decisions.

Collaboration among researchers, health care providers, and technology developers is essential for advancing the field of digital twins in cardiovascular disease management. Data sharing initiatives, research consortia, and interdisciplinary collaborations can accelerate innovation, improve model accuracy, and ensure the ethical and responsible use of digital twin technology [46]. The use of digital twins in health gives rise to ethical dilemmas that require several considerations. Upholding principles such as informed consent, data ownership, and patient autonomy is of utmost importance to guarantee the responsible and ethical integration of digital twins. Establishing well-defined directives and regulatory frameworks becomes imperative for effectively addressing the ethical quandaries stemming from the use of individuals’ personal health data for digital twin modeling. Maintaining reverence for patients’ rights and upholding transparency at all stages is crucial for cultivating a bond of trust between health care providers and their patients.

To date, there has been no comprehensive approach to validating digital twin models. Validating digital twins and simulation models, in general, presents several formidable challenges. While simulations can be validated using retrospective longitudinal data, alternative scenarios are often absent from the ground truth. To instill confidence in the simulation, it becomes imperative to compare the simulated model averages with a separate benchmark. The intricate interplay and diversity within human physiology, disease progression, and individual patient characteristics complicate the task of ensuring an accurate representation of real-world patients. The pursuit of dependable results from digital twins necessitates the implementation of rigorous testing, validation methodologies, and clinical studies. Continual refinement and validation of these models against real-world patient data assume pivotal roles in enhancing their predictive capabilities and expanding their clinical utility [98]. Sensitivity analysis, which model explanations are a subset of, enables the ascription of change in parameters to the outcomes [99]. This empowers a domain expert to identify the most influential factors affecting the outcomes. Subsequently, the domain expert, in our case, the hospital’s operational leadership team, can decide which levers to use to optimize the results. Nevertheless, conducting sensitivity analysis on large-scale simulations becomes computationally challenging due to the numerous parameters involved. [100]. By using machine learning models to train simulated data, we can test the global sensitivity of the model parameters through attribution.

## Conclusions

Digital twins represent a groundbreaking approach to personalized medicine, leveraging digital replicas of patients to optimize diagnostics, treatment strategies, and health care outcomes. Through enhanced understanding of patient health, predictive modeling, digital clinical trials, and remote patient monitoring, digital twins pave the way for more precise, individualized health care interventions. While challenges exist, the continued development and integration of digital twins hold the potential to revolutionize personalized medicine, ushering in an era of tailored treatments and improved patient well-being. Nevertheless, the use of human digital twins gives rise to ethical dilemmas related to informed consent, data ownership, and the potential for discrimination based on health profiles. As we stand on the brink of a new era in medicine, the integration of digital twins offers both exhilarating possibilities and formidable challenges. It is imperative that we, as a medical community, proactively shape ethical guidelines and regulations to harness their full potential while safeguarding patient welfare.

## Abbreviations

AI: artificial intelligence
EHR: electronic health record
IoT: Internet of Things
